# Identification of One Major QTL and a Novel Gene *OsIAA17q5* Associated with Tiller Number in Rice Using QTL Analysis

**DOI:** 10.3390/plants11040538

**Published:** 2022-02-17

**Authors:** Dan-Dan Zhao, Jae-Ryoung Park, Yoon-Hee Jang, Eun-Gyeong Kim, Xiao-Xuan Du, Muhammad Farooq, Byoung-Ju Yun, Kyung-Min Kim

**Affiliations:** 1 Division of Plant Biosciences, School of Applied Biosciences, College of Agriculture and Life Science, Kyungpook National University, Daegu 41566, Korea; qx288mm@naver.com (D.-D.Z.); icd92@naver.com (J.-R.P.); uniunnie@naver.com (Y.-H.J.); dkqkxk632@naver.com (E.-G.K.); mfarooqsr@gmail.com (M.F.); 2 Crop Breeding Division, National Institute of Crop Science, Rural Development Administration, Wanju 55365, Korea; 3 Coastal Agriculture Research Institute, Kyungpook National University, Daegu 41566, Korea; haobingshuaike@hotmail.com; 4 Biosafety Division, National Institute of Agricultural Science, Jeonju 54874, Korea; 5 School of Electronics Engineering, College of IT Engineering, Kyungpook National University, 80 Daehak-ro, Buk-gu, Daegu 41566, Korea

**Keywords:** rice yield, tiller number, quantitative trait locus, auxin, growth stage

## Abstract

Rice tillers are one of the most important traits for the yield and development of rice, although little is known about its mode of inheritance. Tiller numbers were recorded every 7 days a total of nine times, starting 30 days after transplantation. Quantitative trait locus (QTL) based analysis on a set of double haploid population derivatives of a cross between the Cheongcheong and Nagdong varieties identified a major effect of locus RM18130–RM3381 on chromosome 5, which was expressed in eight different growth stages. Within the target region RM18130–RM3381 (physical distance: 2.08 Mb), 61 candidate genes were screened by annotation. Among the candidate genes, *Os05g0230700* (named *OsIAA17q5*), which belongs to the family of auxin-responsive genes, was selected as a target. Auxin promotes cell division and meristem maintenance and is an effective plant regulator which influences plant growth and development by altering the expression of various genes. *OsIAA17q5* is expected to control the number of tillers. The present study provides further understanding of the basic genetic mechanisms that selectively express the control of tiller numbers in different growth stages, as well as provides valuable information for future research aimed at cloning the target gene. These results may contribute to developing a comprehensive understanding of the basic genetic processes regulating the developmental behavior of tiller numbers in rice.

## 1. Introduction

Rice is one of the most valuable food crops and a source of nutrients worldwide. In response to the decline in rice acreage and the growing human population, the main goal of rice breeding programs in recent years has been to increase grain yield [1]. Rice yield is a complex trait that is significantly influenced by various environmental factors and controlled by multiple quantitative trait loci (QTL). Tiller number and their production, which play an important role in determining potential rice yields, are key agronomic traits and components of grain yield [2,3]. However, excessive production of tillers can cause a reduction in yield, because they consume large amounts of nutrients from the main branch during vegetative growth and undergo senescence before maturity without contributing to yield [4]. Moreover, tiller morphology is an important agronomic trait for crop adaptability and phenotypic plasticity. High tillers are best suited to the most favorable growth conditions, whereas low tillers are better suited to stress conditions [5]. In addition, under wet direct seeding conditions, where it is certain that the seed rate competes against weeds, the ideal genotype has less tillering.

Rice tillering is a variable trait that changes over time; during changes in tillering, many biological characteristics play important roles, which could be optimized to manage rice production and improve its genetic foundations. Therefore, parameters such as the optimum tillering time can provide a useful information tool to manage rice production. To optimize these traits, the associated genes must be identified, and little is currently known about the genes that influence these parameters [6].

Using molecular markers mapping the QTL is an effective method to analyze complex traits in crops. QTL mapping has become an important research field in developmental quantitative genetics, that will contribute to achieving a deeper understanding of the genetic basis of quantitative traits used for plant breeding [7]. According to genetic developmental theory, genes are temporally expressed at different growth stages [8]. Moreover, the number of tillers is characterized by dynamic gene expression and is a model trait for studying developmental behavior [2,9]. According to incomplete statistics, more than 200 QTL related to tiller number have been detected in rice on 12 chromosomes, but relatively few of these QTL have been cloned [10]. In addition, the expression of QTL associated with the number of tillers at different growth stages has also previously been reported [2,5,7,11,12,13]. Specifically, Yan et al. (1998) mapped a significant QTL for tiller number on chromosome 1 between markers RZ730 and RZ810 at all measuring stages in rice. Liu et al. (2010) detected 14 QTL that were effective in controlling rice tillers using the conditional analysis method in a single-segment substitution population of rice. Bian et al. (2015) analyzed the developmental behavior of tiller numbers using QTL analysis, detecting 21 QTL in an introgression line population. Similarly, using a genome-wide association study (GWAS) identified 38 ETN-associated QTL, among which 4 were localized with *NAL1*, *OsWRKY74*, *OsAAP1*, and *DWL2*, and showed that Hap5 of *OsAAP1*, Hap3 and Hap6 of *DWL2*, and Hap3 and Hap4 of *WRKY74* are favorable alleles for effectively controlling tiller number and are involved in the regulation of rice tillering [14]. Another recent study that used GWAS identified that 23 loci associated with tiller number variations (LATNs) are significantly correlated with the differences. Among the 23 LATNs, 8 are co-localized with previously cloned tiller number genes, and the remaining 15 LATNs are novel. DNA sequence analysis of the 15 novels LATNs led to the identification of 5 candidate genes using the accessions with extreme tiller number phenotypes [15]. Although there are many studies on the QTL analysis of tiller numbers, the genetics of tillering control has not yet been thoroughly studied. In rice, yield-related traits are influenced by multiple environmental and genetic factors, which determine continuous variations in phenotypes and form a genetically controlled phenotypic network of multiple loci [16]. The integration of multiple reports on QTL could improve understanding of the genetic basis of complex quantitative traits [17]. This is important for elucidating the genetic mechanisms behind the effect of tiller numbers on rice yield, in order to develop better genotypes adapted to the growing environment [5].

In this study, we analyzed the developmental behavior of rice tillers using a “Cheongcheong” × “Nagdong” double haploid (CNDH) line. The CNDH lines have been cultivated in a generational process for over 10 years at the Kyungpook National University of Gunwi-gun test field, and it has been used as a bridging parent [18,19]. The aim of this study was to detect the QTL controlling tiller number in the CNDH lines and to explore the dynamics of QTL associations during the whole ontogeny. To further elucidate the genetic mechanisms that control the tiller number in rice, this trait was investigated at nine different growing stages for two consecutive years. To identify stable and novel genomic regions associated with tiller number, the detected QTL were compared with loci previously reported in the literature, which may be further exploited for precise introgression in target cultivars.

## 2. Results

### 2.1. Phenotype Evaluation

**Figure 1:**
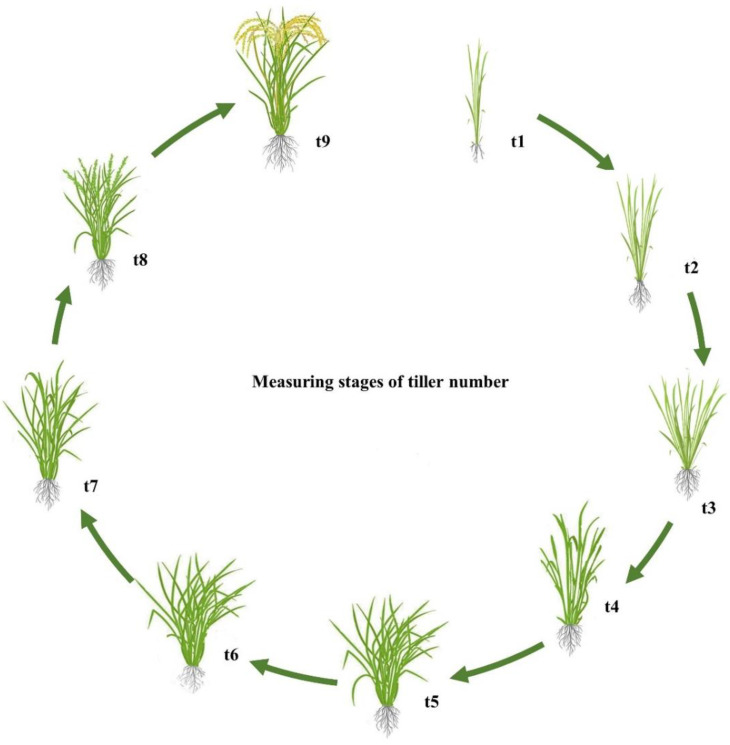
Tiller number measuring stages. The tiller number of each plant was investigated every 7 days, starting 30 days after planting the seedling into the field (stages are denoted as t1 to t9).

**Figure 2:**
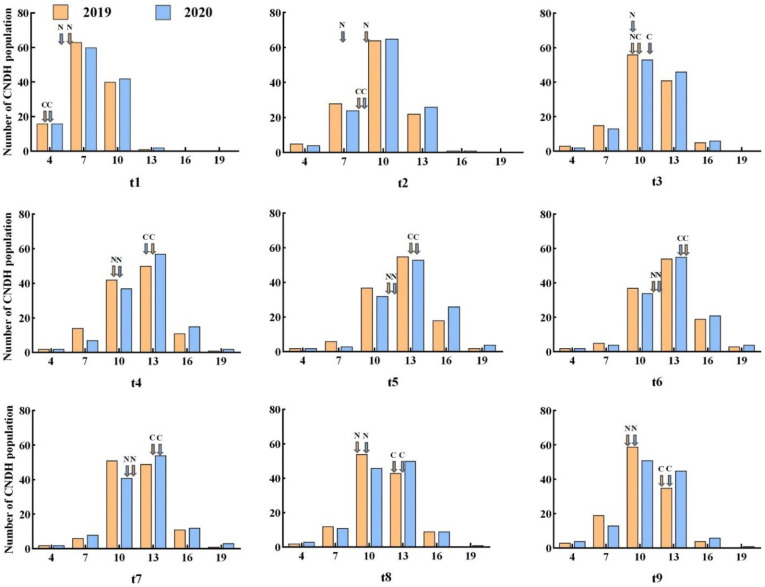
Tiller number averaged over the CNDH population at different growth stages. t1 to t9 refers to the tiller number at each measuring stage; the measurement intervals were set as 7 days between stages. (C) Cheongcheong; (N) Nagdong.

**Figure 3:**
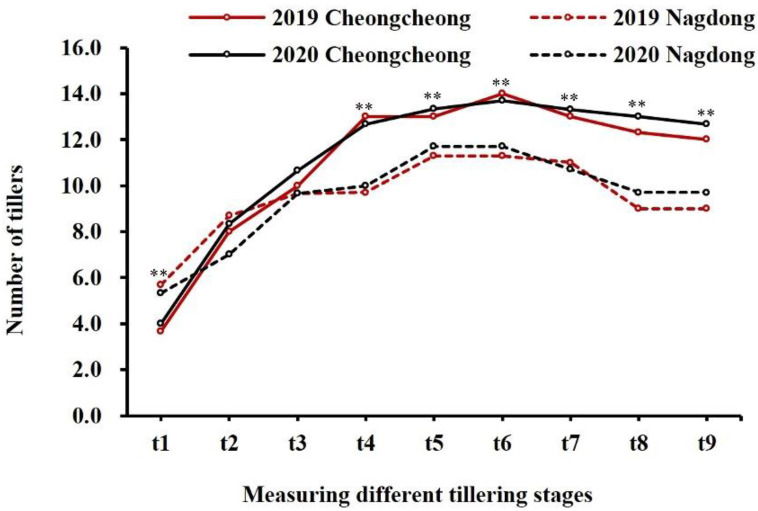
Weekly variation in the tiller number of Cheongcheong and Nagdong. The tiller number of each plant was investigated every 7 days, starting 30 days after planting the seedling into the field (denoted as t1 to t9). Values are means ± SD (*n* = 3). ** *p* < 0.01 (two-tailed Student’s t-test).

**Figure 4:**
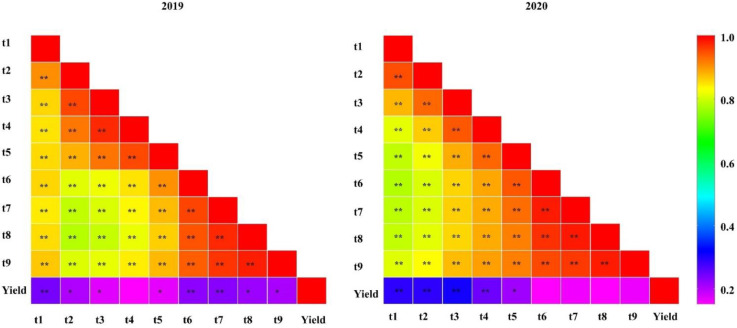
Analysis of the correlation between tiller number at each measuring stage and yield in the CNDH population in 2019 and 2020. ** Correlation is significant at the 0.01 level (2-tailed); * correlation is significant at the 0.05 level (2-tailed).

The tiller numbers counted at different stages are shown in Figure 1. The average tiller numbers of the 120 CNDH lines at nine stages in 2019 and 2020 are shown in Figure 2. Based on these data, collected over two years, a comparative analysis of the tiller number at each stage was conducted and is shown in Table S1. In stage t1, Nagdong (5.7) had more tillers than Cheongcheong (3.7), but upon reaching the maximum tillering stage, the former exhibited a decline due to the death of young tillers. The CNDH lines reached the maximum number of tillers at stage t6, with a minimum number of tillers of four. A pattern of a continuous distribution of tiller number was observed in different growth stages, indicating that this was a typical quantitative trait and that it was controlled by polygenes. The number of tillers in the Cheongcheong and Nagdong parental lines measured at different growth stages are presented in Figure 3. At t1, t4, t5, t6, t7, t8, and t9 stages, Cheongcheong and Nagdong have significant differences with each other in both years. The number continually increased, reaching the highest tillering level at stage t6. Subsequently, it was reduced to the final effective tiller number. The rapid increase in tiller number appeared before stage t4, after which the rate gradually slowed to the highest tillering stage. The curves show slight differences between Cheongcheong and Nagdong in 2019 and 2020, and their variation may be attributed to differences in gene expression that characterize the two rice varieties. Correlation analysis between the tiller number at each stage and yield showed a highly significant correlation in 2019, except for at stage t4. However, each stage from t1 to t5 in 2020 presented a positive correlation with the yield (Figure 4). The slight variation between the two years might have been due to environmental factors.

### 2.2. Detection of QTL for Tiller Number

**Figure 5:**
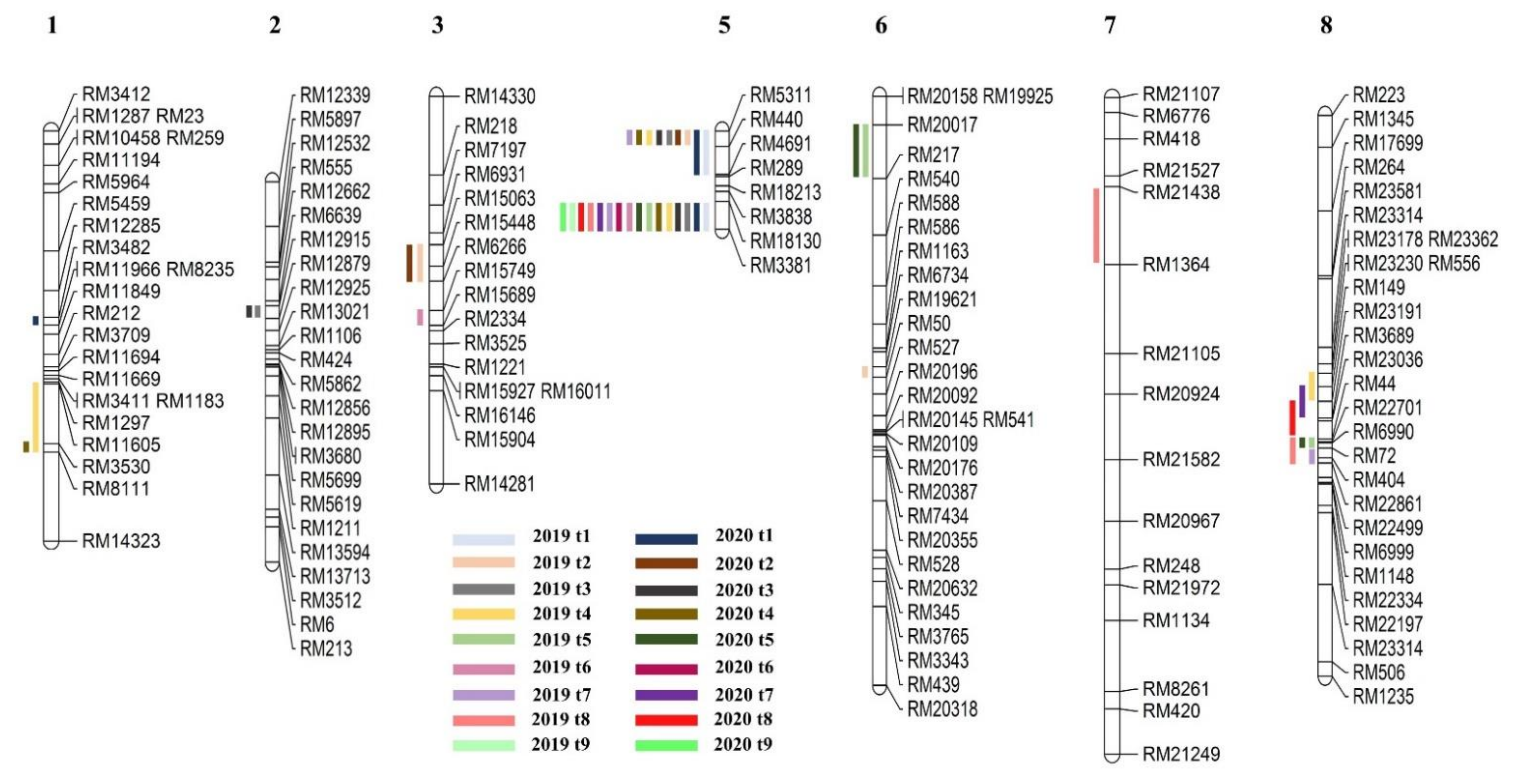
Chromosomal location of QTL related to tiller number at different growth stages in the CNDH lines.

The analysis was carried out for each growth stage in 2019 and 2020. Detailed information on the QTL detected in the nine different stages is shown in Table S2 and Figure 5. A total of 44 QTL influencing the number of tillers were detected in the CNDH population, which were located on chromosomes 1, 2, 3, 5, 6, 7, and 8. Specifically, 24 and 20 QTL were detected in 2019 and 2020, respectively. Overall, three QTL were detected on chromosomes 1, 3, and 6, whereas two, one, and seven QTL were detected on chromosomes 2, 7, and 8, respectively, and 25 QTL were detected on chromosome 5. In particular, 16 of these could be detected repeatedly in different growth stages in both years; thus, they were considered as target QTL. They explained 22–36% of the phenotypic variance and had LOD values between 2.82 and 5.94. The positive alleles of 12 QTL were derived from Cheongcheong, whereas those of the remaining 4 QTL were from Nagdong. Therefore, it is inferred that the genes controlling the number of tillers may play different roles in different growth stages, but the target interval RM18130–RM3381 on chromosome 5 is the main control region.

### 2.3. Gene Screening from a Consistent QTL Marker Interval

**Figure 6:**
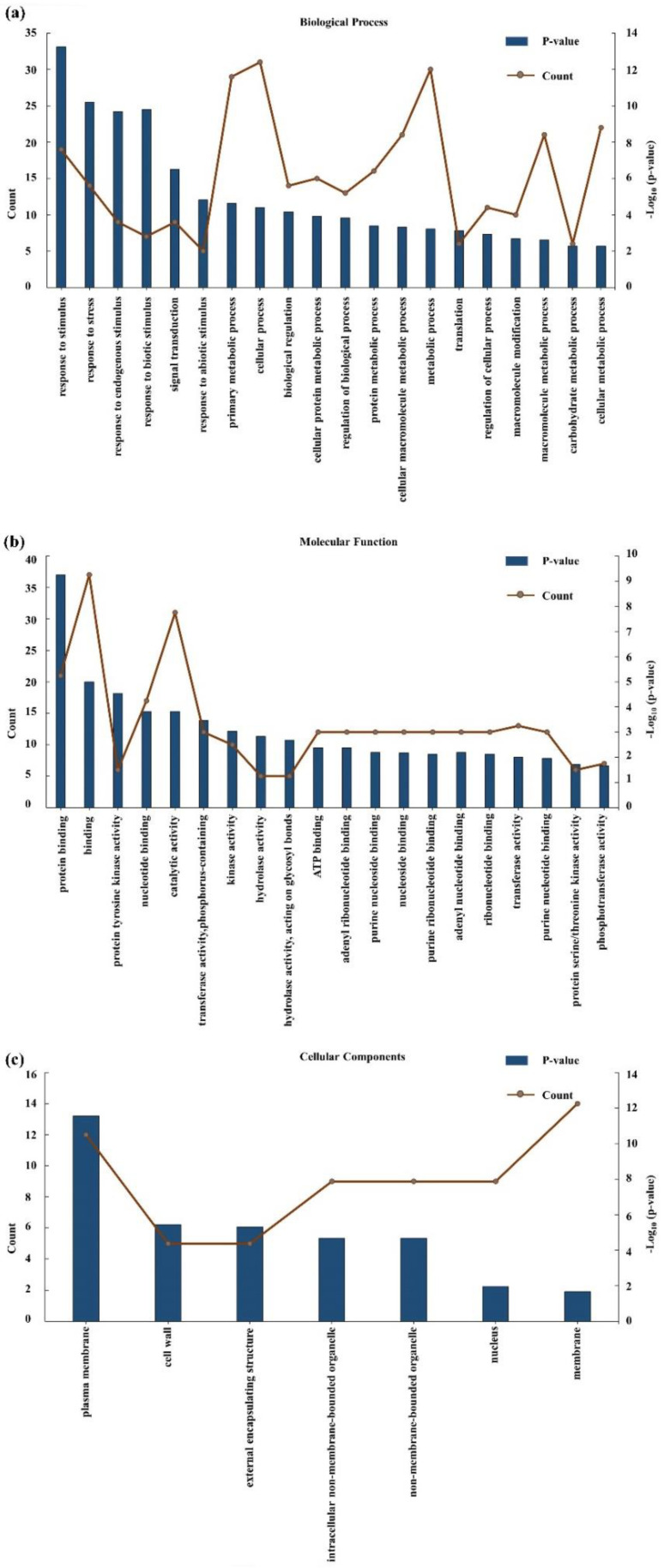
Gene ontology annotation of the QTL target region RM18130–RM3381. (**a**) Biological Process. (**b**) Molecular Function. (**c**) Cellular Component.

**Figure 7:**
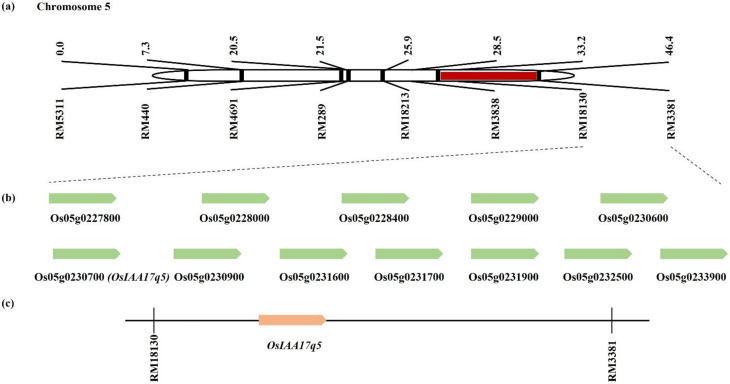
Physical mapping of the genes related to tiller number. (**a**) Target interval RM18130–RM3381 on chromosome 5. (**b**) In this case, 12 representative candidate genes located near the target gene *OsIAA17q5*, between RM18130 and RM3381 on chromosome 5. (**c**) Screening of *OsIAA17q5*, among the candidate genes, as a target gene-related to tiller number.

In the present study, the candidate genes within the interval RM18130–RM3381 on chromosome 5 were screened using the database available from RiceXpro https://ricexpro.dna.affrc.go.jp/ (accessed on 14 January 2022). A total of 85 unique genes were identified in this interval. AgriGO was used as a reference database to summarize the possible functional classifications of rice gene identifiers between RM18130 and RM3381 on chromosome 5. Significant GO terms for 85 unique genes within the marker interval were identified, and the 20, 20, and 7 top-ranking significant terms were selected for “Biological Process,” “Molecular Function,” and “Cellular Component,”, respectively (Figure 6). The most significantly enriched GO terms associated with “Biological Process” were a response to stimulus, stress, endogenous stimulus, biotic stimulus, and signal transduction; the terms most frequently associated with “Molecular Function” included protein binding, protein tyrosine kinase activity, nucleotide-binding, and catalytic activity; and finally, the most frequent terms for “Cellular Component” were plasma membrane, cell wall, and external encapsulating structure. Overall, a total of 61 candidate genes at region RM18130–RM3381 were selected based on the available sequence annotations (Table S3). Among them, *Os05g0230700*—which is similar to the auxin-responsive protein IAA17—was selected as the target gene (Figure 7).

### 2.4. Homology Sequence and Phylogenetic Tree Analysis of OsIAA17q5

**Figure 8:**
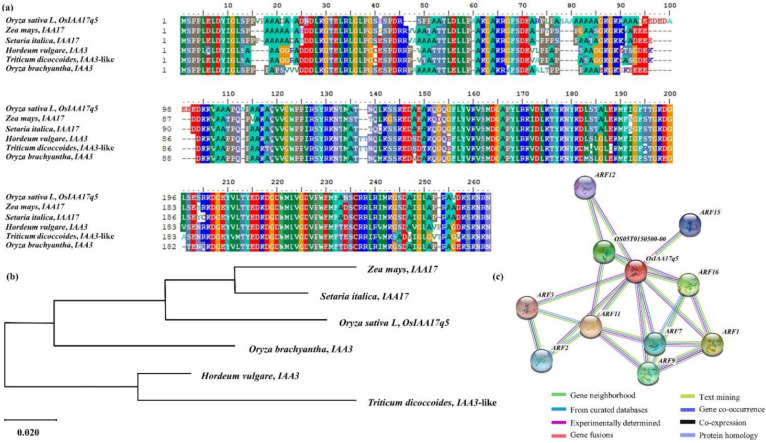
Sequence analysis of *OsIAA17q5*. (**a**) Comparison of the protein sequences of *OsIAA17q5* homologous genes; a very high similarity was found in *Zea mays*, *Setaria italica*, *Hordeum vulgare*, *Triticum dicoccoides*, and *Oryza brachyantha*. (**b**) Phylogenetic tree used to analyze *OsIAA17q5* and its homologous genes. The phylogenetic tree was constructed with 1000 bootstrap replicates using the parsimony method. (**c**) Protein interaction of *OsIAA17q5*. The gene interacts with OS05T0150500-00, ARF1, ARF2, ARF3, ARF7, ARF9, ARF11, ARF12, ARF15, and ARF16.

The target gene *OsIAA17q5* was screened for tiller number effects in eight different growth stages during the QTL analysis for the 120 CNDH lines. Furthermore, using the NCBI database, BLAST analyses showed that *OsIAA17q5* has a very similar sequence to that of IAA17 in *Zea mays* and *Setaria italica*, and that of IAA3 in *Hordeum vulgare*, *Triticum dicoccoides*, and *Oryza brachyantha* (Figure 8a). The phylogenetic tree analysis confirmed the genetic similarity between the *OsIAA17q5* of *Setaria italica*, *Zea mays*, *Oryza brachyantha*, *Hordeum vulgare*, and *Triticum dicoccoides* (Figure 8b). Moreover, using the domain of *OsIAA17q5* to predict the functional partners, it was found that *OsIAA17q5* exhibited interactions with 10 different proteins (OS05T0150500-00, ARF1, ARF2, ARF3, ARF7, ARF9, ARF11, ARF12, ARF15, and ARF16) (Figure 8c).

### 2.5. Comparison of Candidate Gene Expression Level of CNDH Lines and Parental Lines

**Figure 9:**
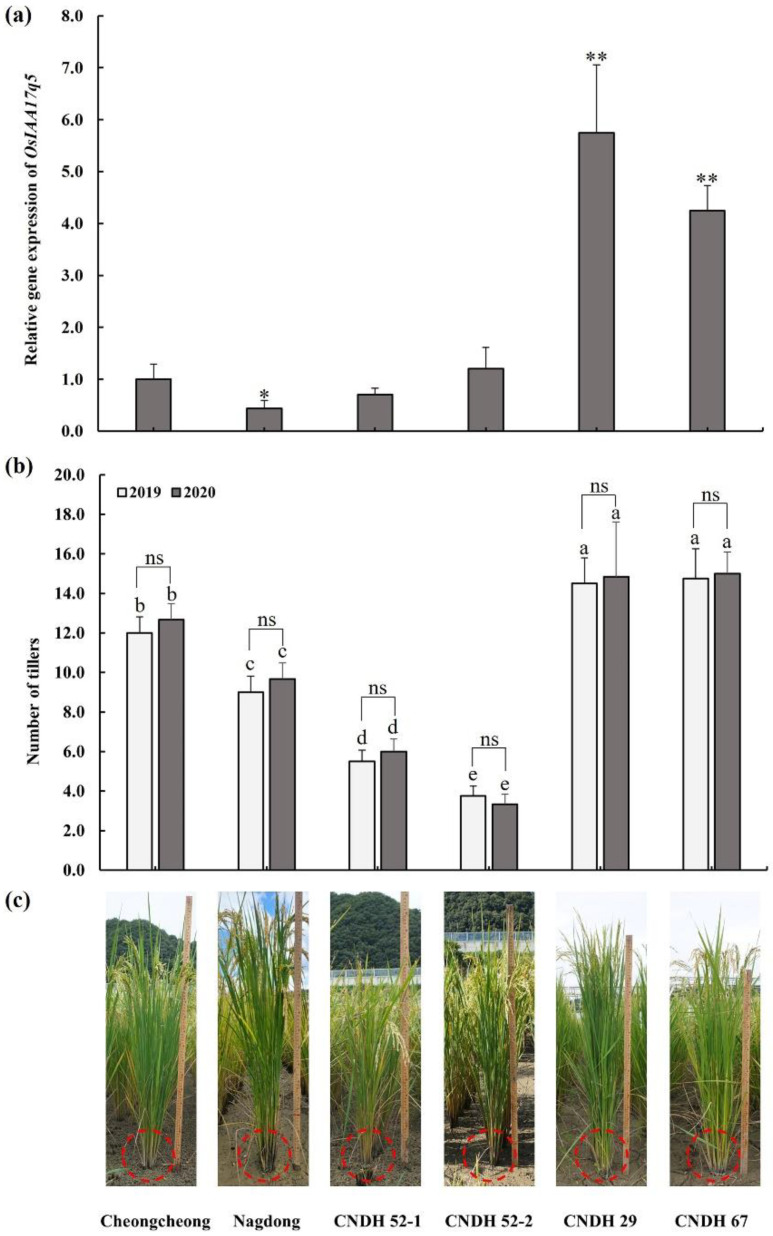
Analysis of relative expression levels for *OsIAA17q5* and morphological characters in the CNDH lines and parental lines. (**a**) The expression of *OsIAA17q5* in Cheongcheong, Nagdong, CNDH 52-1, CNDH 52-2, CNDH 29, and CNDH 67. Values are means ± SD (*n* = 3). * *p* < 0.05, ** *p* < 0.01 (two-tailed Student’s t-test). (**b**) Column diagrams showing the number of tillers in 2019 and 2020. ns, no significant difference, (two-tailed Student’s t-test). Statistical comparisons were made using one-way variance analysis/Duncan’s multiple range test. The different letters above the column represent a statistically significant difference at *p* < 0.05. Values are means ± SD (*n* = 6) (**c**) Morphological characters in the CNDH lines and parental lines. (Measuring ruler = 1m).

To compare the expression levels of candidate gene *OsIAA17q5*, we used quantitative real-time analysis of *OsIAA17q5*, CNDH 52-1, and CNDH 52-2, which have less tillers, and CNDH 29 and CNDH 67, which have more tillers and compare with their parental lines Cheongcheong and Nagdong. The expression level of *OsIAA17q5*, Cheongcheong had higher expression than Nagdong. Expression levels of CNDH 52-1 and CNDH 52-2 have no significant difference with Cheongcheong. However, CNDH 29 and CNDH 67 showed a significant difference in the relative expression levels with Cheongcheong (Figure 9a). Furthermore, no significant difference was observed in tiller number between 2019 and 2020 among all lines. However, the statistical analysis between each genetic material shows significant difference in CNDH29 and CNDH67 compared to parental lines (Cheongcheong and Nagdong) but show no significant difference with each other, while the CNDH52-1 and CNDH52-2 show highly significant differences compare to Cheongcheong and Nagdong with less tillers (Figure 9b).

## 3. Discussion

Tiller number is one of the most important agronomic traits for rice grain production [5], but it is particularly complex and is affected by both genetic and environmental factors. In recent years, with the development of QTL mapping technology and molecular marker maps, the accurate QTL mapping of tiller numbers has been applied to several species [13,20,21]. Japanese breeders identified that the gene *Ltn(t)* controlled tiller numbers; it was responsible for a low number of tillers in the Aikawa1 rice variety and was detected on chromosome 8 near the simple sequence repeat marker RM264 [22]. Moreover, the *MONOCULM1 (MOC1)* gene on chromosome 6 is an important gene for the control of tiller number, and the mutant plant of *MOC1*, due to defective tillering bud formation, only had the main stem, without any tillers [23]. Previous studies have detected a major QTL (tn1–4) at all measuring stages on chromosome 1 within the interval between markers RZ730 and RZ810, it means that in each developmental stage, the QTL effect was different, but there was one consistent region in developmental stages [2].In addition, *OsCCD7*—an orthologous *AtMAX3/CCD7* gene in rice—was identified as a candidate gene related to tillering and dwarfing [24]. However, map-based cloning of the *D3* gene—which is an F-box leucine-rich repeat protein orthologous to *Arabidopsis MAX2/OPE9*, that controls axillary bud activity in monocotyledon and dicotyledon plants—indicates that bud activity controls rice tillering and shoot branching [25].

Therefore, in the present study, the DH line and their recipient parent were used as experimental material for the genetic analyses of tiller number. Since 2010, the DH line has been reproduced every year at the paddy field, and it has become a bridge population that exhibits a variety of traits. Moreover, each line, which represents a variety of traits, is constructed and suitable for QTL analysis [26,27,28,29,30]. QTL analysis was performed on the phenotypic values of tiller number at nine different growth stages, and the genetic variations and corresponding QTL for tiller number at each stage were determined. The number of QTL varied according to the different detection stages. It can be speculated that the variation in genetic factors leads to changes in tiller number dynamics. In this study, during the two years, a total of 44 QTL were detected from which 16 QTL were located on chromosome 5 between interval RM18130-RM3381. Similarly, although the QTL detected in the nine different measuring stages were different, the numbers and functions of QTL affecting the number of tillers were different in different periods [2]. Furthermore, the significant target interval RM18130–RM3381 contained 61 related genes. Among them, we focused on candidate gene *OsIAA17q5*, which is similar to the auxin-responsive protein IAA17.

As a plant hormone, auxin regulates many key growth and development processes in rice [31]. Specifically, it is involved in coleoptile elongation [32], and it promotes cell division, meristem maintenance, enlargement, and differentiation [33,34,35]. Auxin is synthesized in plant cells and is actively transported between cells by polar transport. Additionally, this hormone mediates root growth, tiller number, leaf shape growth, and grain size in rice [36]. Similarly, previous studies have revealed that auxin-related genes play a key role in controlling tillering and plant architecture in rice. According to Chen et al. (2012), the knockdown of *OsPIN1* leads to an increase in both the number and angle of tillers. Interestingly, overexpression of *OsPIN2* and *OsPIN3t* also increases in tiller number and angle [31]. The BLAST analysis results for *OsIAA17q5*, obtained using the NCBI database, showed a nucleotide sequence similarity with IAA17 and IAA3. *OsIAA17q5* exhibited the highest similarity with IAA17 of *Zea mays* and *Setaria italica*. This protein participates in determining the typical phenotypes associated with auxin signaling regulation, such as hypocotyl elongation, root orientation, root hair, and adventitious root formation [37,38]. In summary, the roles of Aux/IAA17 and IAA3 in plant growth and development processes are related to gravitropism, apical dominance, leaf morphology, lateral branch, and hypocotyl elongation [39]. In addition, *OsIAA17q5* interacts with the proteins OS05T0150500-00, ARF1, ARF2, ARF3, ARF7, ARF9, ARF11, ARF12, ARF15, and ARF16, which are all related to auxin response factors. They play a role in auxin-activated signaling pathways [40]. Moreover, many plant researchers have reported the role and involvement of Aux/IAA gene families in plants growth development, especially in tiller outgrowth, as Harin et al., 2015 reported that a knock-down mutant of *OsIAA6* showed abnormal tiller outgrowth, apparently due to the regulation of the auxin transporter *OsPIN1* and the rice tillering inhibitor *OsTB1*, and their results confirm that the *OsIAA6* gene is involved in drought stress responses and the control of tiller outgrowth [41]. Similarly, Jin et al., 2016 reported that transgenic rice plants overexpressing *OsIAA10* were substantially stunted compared to non-transgenic control plants or transgenic plants overexpressing the wild-type *OsIAA10*, and these transgenic plants developed more tillers, shorter crown roots, and lower fertility rates, while overexpression of wild-type *OsIAA10* caused a moderately increased tillering and dwarf phenotypes [42].

To evaluate the role of our target gene *OsIAA17q5* at t9 stage, we checked the expression level of *OsIAA17q5* in the parental lines (Cheongcheong and Nagdong), more tiller lines (CNDH29 and CNDH67), and less tiller lines (CNDH52-1 and CNDH52-2). The current results show the expression level of *OsIAA17q5* was significantly high in the CNDH29 and CNDH67 compared to CNDH52-1, CNDH52-2, and parental lines. Similarly, Zhang et al., 2009 reported that reduced in IAA maxima at the shoot apexes or basal nodes are due to the conversion of free IAA to its inactive form in the *TLD1/OsGH3.13* and its overexpression also resulted in excessive tillering, this finding also supports the suggested role of IAA homeostasis in the control of rice tillering [43]. Xu et al., 2005 reported that *OsPIN1* closer to the *PIN1* family (the main auxin transporter transporting auxin towards the root tip in the vascular tissue) was expressed in the vascular tissues and root primordial such as *AtPIN1*, and therefore can play important role in auxin-dependent adventitious root emergence and tillering [44].

In this study, *OsIAA17q5* was finally selected from the 61 candidate genes associated with target region RM18130-RM3381. The QTL influencing the tiller number which was screened in the present study can be used in breeding programs to develop environmentally suitable cultivars. The *OsIAA17q5* gene identified in this study needs further experimentation to elucidate its molecular cloning and characterization. Additionally, in future studies, the identification of this gene will be useful to clarify the mechanisms of tiller development.

## 4. Materials and Methods

### 4.1. Plant Material and Mapping Population

In the present study, Cheongcheong, Nagdong, and 120 double haploid lines—derived from a cross between the *Indica* variety Cheongcheong and the *Japonica* variety Nagdong—were used to analyze tiller numbers [19]. The genetic map was constructed using the DH lines, which was developed by anther culture [45]. The DH lines map was built using 788 SSR markers. Polymorphism was identified in 423 SSR markers by polymorphism analysis. Among them, 222 SSR markers were selected based on the codominant genes amplified through PCR amplification [19]. The CNDH lines genetic map covered a total genetic distance of 2121.7 cM, with an average distance between markers of 10.6 cM [45]. Mapmaker version 3.0 was used to draw the genetic map, and the markers were evenly distributed on the 12 rice chromosomes [46].

### 4.2. Field Experiment Design and Evaluation of Tiller Number

The present experiments were conducted at the Gunwi-gun experimental field (Gyeongbuk, Korea), located at latitude ~36°11′ N and longitude ~128°64′ E, from May to October in 2019 and 2020. Before sowing, seeds were surface sterilized with 25% prochloraz (Hankook Samgong, Seoul, Korea) and soaked in tap water at 33 °C for 3 days in an incubator in the dark. Once germinated, the seeds were sown in a seedling bed, and then seedlings were planted into the experimental field after 30 days. The seedlings were planted in six rows for each CNDH line and their parents, with 24 seedlings in each row; the planting density was 0.3 m × 0.15 m. A randomized completely block design was used for all lines. Field experiments were managed in accordance with regular agricultural practices. To control pests and diseases, insecticides and herbicides were used, following the standard cultivation methods established by the Rural Development Administration. The amounts of N, P_2_O_5_, and K_2_O fertilizers used were 9, 4.5, and 5.7 kg per 10 ha, respectively. Subsequently, 30 days after transplantation, the number of tillers in each DH line was measured every 7 days, and six plants per line were measured until plant heading (the plants were fixed through all measuring stages). A total of nine different growth periods were measured, and the number of tillers was recorded continuously throughout (denoted as from t1 to t9). The average tiller numbers of six plants in each plot were used as the data for analysis. The measurement of the grain yield was based on the following formula: Y (grain yield, t/ha) = N (spikelet number/m^2^) × W(1000-grain weight, g) × F (filled spikelet) × 10^−5^ [47].

### 4.3. Statistical and QTL Analysis

The average measurements of the number of tillers, conducted in six replicates, were used for subsequent analyses. Statistical analysis was performed through calculations of the mean and standard deviation. The frequency distribution graph was plotted and analyzed using GraphPad Prism (version 8.0.2). Pearson’s correlation was performed in SPSS (version 25). The Student’s t-test and one-way variance analysis were used to evaluate the significance of the difference. QTL analysis of the tiller number at different growth stages was conducted using Windows QTL Cartographer 2.5 software, with the composite interval mapping method [48]. QTL denominations were based on the nomenclature proposed by McCough and Doerge [49]. The odds score threshold of LOD was set at 2.5 [48]. In order to run the QTL analysis, all the required data—including chromosome numbers, genetic distance, marker labels, genotyping data, and target trait values—were entered.

### 4.4. Prediction of Candidate Genes

Based on QTL analysis results, RiceXpro (https://ricexpro.dna.affrc.go.jp/ (accessed on 14 January 2022) [50] and RAP-DB (https://rapdb.dna.affrc.go.jp/ (accessed on 14 January 2022) were used to further screen the candidate genes and to create a physical map. ORFs were found in SSR markers, and candidate genes were annotated and classified by gene function. The functions of candidate genes were identified through gene ontology (GO) enrichment analysis, using the agriGO tool (http://bioinfo.cau.edu.cn/agriGO/ (accessed on 14 January 2022) [51]. For multiple homologous sequences, the NCBI and BioEdit 7.0 comparison was used [52]. The STRING (version 11.0) (https://string-db.org/ (accessed on 14 January 2022) database was used for the analysis of the protein–protein interaction/association network [53].

### 4.5. Analysis of Expression Levels of OsIAA17q5

Among the CNDH lines, CNDH 52-1 and CNDH 52-2 (less tillers line), CNDH 29 and CNDH 67 (more tillers line), and their parental lines, the rice leaves were sampled at the t9 stage. The total RNA extraction was carried out using the RNeasy Plant Mini Kit (QIAGEN, Hilden, Germany) according to the manufacturer’s instructions. The RNA was diluted to a final concentration of 100 ng/µL using Rnase-free water and cDNA was synthesized 100 ng RNA as a template using qPCRBIO cDNA Synthesis Kit (PCR Biosystems, USA). The cDNA was used as a template in the StepOnePlus™ Real-Time PCR System machine (Thermo Fisher Scientific, Seoul, Korea) using the 2X Real-time PCR Master Mix (including SYBR^®^ Green I BioFACT™, Daejeon, Korea) along with 100 ng of template DNA and 10 nM of each primer to a final volume of 20 µL. 40 cycles of a two-step PCR reaction were established followed by the under conditions: At 95 °C for 15 min of polymerase activation, at 95 °C for 15 s of denaturation, and annealing and extension at 60 °C for 30 s. The *OsActin* was used as an internal reference gene. Each reaction was repeated three times, and the primer used in this study is listed in Table S4.

## 5. Conclusions

Our results indicate that the *OsIAA17q5* gene, screened by the QTL analysis in different growth stages, has a potential role in controlling the number of tillers. Further studies are needed to clarify its molecular mechanisms in rice. Moreover, the newly discovered QTL regulating tiller number may provide a potential new approach to developing ideal rice genotypes. Finally, these results also provide a basis for cloning QTL that significantly contributes to influencing the tiller number in rice.

## Data Availability

The data presented in this study are available on request from the corresponding author.

## Supplementary Materials

The following are available online at https://www.mdpi.com/article/10.3390/plants11040538/s1, Table S1: Phenotypic values of tiller number in the CNDH lines and its parents, “Cheongcheong” and “Nagdong,” at nine different growth stages. Table S2: QTL related to tiller number at different growth stages in the CNDH lines. Table S3: Candidate genes from the target interval RM18130–RM3381 on chromosome 5 related to tiller number in the CNDH lines. Table S4: List of primers used in this study.
