# Supplementary material for: Identification of One Major QTL and a Novel Gene OsIAA17q5 Associated with Tiller Number in Rice Using QTL Analysis
Source: Plants (Basel). 2022 Feb 17;11(4):538. doi: 10.3390/plants11040538 (PMC8875189; doi:10.3390/plants11040538)
Supplement: Supplementary file 1 [file plants-11-00538-s001.zip › plants-1576416-supplementary.pdf]

Table S1. Phenotypic values of tiller number in the CNDH lines and its parents, "Cheongcheong" and "Nagdong," at nine different growth stages.

Stage	Year	Parents		DH Population				
		Cheongcheong	Nagdong	Max.	Min.	Mean	Skewness	Kurtosis
t1	2019	3.70±0.70	5.70±0.70	12.00	3.00	7.71±1.76	-0.43	-0.10
	2020	4.00±0.60	5.30±0.50	12.00	3.00	7.64±1.94	-0.26	-0.37
t2	2019	8.00±0.60	8.70±0.70	15.20	3.00	9.64±2.22	-0.45	0.29
	2020	8.30±0.90	7.00±0.60	15.70	3.30	9.82±2.22	-0.30	0.21
t3	2019	10.00±0.60	9.70±0.90	17.00	3.30	10.58±2.40	-0.32	0.58
	2020	10.70±0.50	9.70±0.50	16.30	3.30	10.96±2.34	-0.39	0.68
t4	2019	13.00±0.60	9.70±0.90	17.70	3.30	11.41±2.53	-0.35	0.54
	2020	12.70±1.10	10.00±0.60	17.70	4.00	12.02±2.45	-0.47	0.77
t5	2019	13.00±0.60	11.30±0.50	18.00	3.80	11.41±2.53	-0.35	0.54
	2020	13.30±0.70	11.70±0.70	18.30	4.30	12.02±2.45	-0.47	0.77
t6	2019	14.00±1.00	11.30±0.50	19.30	4.00	12.37±2.59	-0.29	1.23
	2020	13.70±0.50	11.70±0.50	20.00	4.00	12.56±2.74	-0.29	0.91
t7	2019	13.00±0.60	11.00±0.60	17.70	3.70	11.59±2.46	-0.34	0.47
	2020	13.30±0.50	10.70±0.50	19.30	3.70	11.95±2.69	-0.27	0.70
t8	2019	12.30±0.50	9.00±1.20	15.00	3.70	11.06±2.36	-0.45	0.22
	2020	13.00±0.60	9.70±0.90	18.50	3.30	11.39±2.57	-0.38	0.58
t9	2019	12.00±0.60	9.00±0.80	15.00	3.30	10.59±2.29	-0.41	0.36
	2020	12.70±0.50	9.70±0.70	17.70	3.30	10.95±2.46	-0.33	0.43

Data are presented as mean ± standard deviation. The tiller number of each plant was investigated every 7 days from 30 days after transplanting (denoted as t1, t2, t3, t4, t5, t6, t7, t8, and t9, respectively).

Table S2. QTL related to tiller number at different growth stages in the CNDH lines.

Stage [a]	Year	Chromosome	Marker Interval [b]	LOD	Add. Effect [c]	R^2 [d]	Increasing Effect [e]
t1	2019	5	RM5311-RM4691	2.84	-0.84	0.35	Nagdong
		5	RM18130-RM3381	3.60	0.92	0.32	Cheongcheong
	2020	1	RM3482-RM11966	2.57	0.75	0.33	Cheongcheong
		5	RM5311-RM4691	3.21	0.90	0.34	Cheongcheong
		5	RM18130-RM3381	3.28	-0.89	0.33	Nagdong
t2	2019	3	RM15063-RM6266	4.26	1.14	0.34	Cheongcheong
		5	RM5311-RM440	4.25	1.13	0.35	Cheongcheong
		6	RM19621-RM50	2.53	-0.81	0.34	Nagdong
	2020	3	RM15063-RM6266	3.92	1.12	0.33	Cheongcheong
		5	RM5311-RM440	4.42	1.17	0.33	Cheongcheong
t3	2019	2	RM12915-RM12879	2.72	1.19	0.36	Cheongcheong
		5	RM5311-RM440	3.86	1.09	0.39	Cheongcheong
		5	RM18130-RM3381	3.48	-0.96	0.36	Nagdong
	2020	2	RM12915-RM12879	2.74	1.25	0.36	Cheongcheong
		5	RM5311-RM440	3.67	1.07	0.36	Cheongcheong
		5	RM18130-RM3381	3.15	-0.97	0.36	Nagdong
t4	2019	1	RM1297-RM8111	3.58	0.89	0.37	Cheongcheong
		5	RM5311-RM440	3.45	-0.81	0.37	Nagdong
		5	RM18130-RM3381	3.87	0.86	0.36	Cheongcheong
		8	RM23230-RM23191	3.42	0.85	0.37	Cheongcheong
	2020	1	RM3530-RM8111	2.51	0.74	0.34	Cheongcheong
		5	RM5311-RM440	3.02	-0.78	0.34	Nagdong
		5	RM18130-RM3381	3.49	0.85	0.34	Cheongcheong
t5	2019	5	RM18130-RM3381	4.48	1.06	0.33	Cheongcheong
		6	RM20017-RM217	2.68	0.89	0.35	Cheongcheong
		8	RM44-RM72	3.26	0.83	0.34	Cheongcheong
	2020	5	RM18130-RM3381	4.50	1.06	0.33	Cheongcheong
		6	RM20017-RM217	2.86	1.00	0.37	Cheongcheong
		8	RM44-RM72	2.80	0.76	0.33	Cheongcheong
t6	2019	3	RM15749-RM15689	2.61	1.08	0.22	Cheongcheong
		5	RM18130-RM3381	3.71	1.31	0.22	Cheongcheong

	2020	5	RM18130-RM3381	3.59	1.31	0.22	Cheongcheong
t7	2019	5	RM5311-RM440	2.54	0.86	0.33	Cheongcheong
		5	RM18130-RM3381	2.71	-0.80	0.31	Nagdong
		8	RM72-RM22861	2.56	0.78	0.32	Cheongcheong
	2020	5	RM18130-RM3381	3.59	0.95	0.27	Cheongcheong
		8	RM149-RM3689	2.95	0.96	0.34	Cheongcheong
t8	2019	5	RM18130-RM3381	2.82	0.75	0.29	Cheongcheong
		7	RM21438-RM1364	2.52	0.94	0.37	Cheongcheong
		8	RM44-RM22861	3.98	0.87	0.32	Cheongcheong
	2020	5	RM18130-RM3381	3.19	0.80	0.29	Cheongcheong
		8	RM23191-RM44	3.55	0.83	0.31	Cheongcheong
t9	2019	5	RM18130-RM3381	5.94	1.11	0.29	Cheongcheong
	2020	5	RM18130-RM3381	5.76	1.13	0.30	Cheongcheong

[a] The tiller number of each plant was investigated every 7 days from 30 days after transplanting (denoted as t1, t2, t3, t4, t5, t6, t7, t8, and t9, respectively).

[b] Marker Interval are those within the significance threshold on each border of the QTL range.

[c] Additive effect.

[d] Phenotypic variation explains each QTL.

[e] Increase effect is the source of the allele causing an increase in the measured traits.

Table S3. Candidate genes from the target interval RM18130-RM3381 on chromosome 5 related to tiller number in the CNDH lines.

Locus ID	Description
Os05g0223000	Similar to Calmodulin-related protein (Fragment).
Os05g0223200	Similar to Glycine-rich RNA-binding protein GRP2A.
Os05g0223300	Similar to RNA-binding protein.
Os05g0224700	RNA polymerase Rpb7, N-terminal domain containing protein.
Os05g0224800	Similar to Modification methyltransferase, cytosine-specific.
Os05g0225800	Similar to Szp protein.
Os05g0227600	Quinonprotein alcohol dehydrogenase-like domain containing protein.
Os05g0227800	Homeodomain-like containing protein.
Os05g0228000	Purine and other phosphorylases, family 1 protein.
Os05g0228400	Helix-loop-helix DNA-binding domain containing protein.
Os05g0229000	D111/G-patch domain containing protein.
Os05g0230600	Protein of unknown function DUF1620 domain containing protein.
Os05g0230700	Similar to Auxin-responsive protein (Aux/IAA) (Fragment).
Os05g0230900	Similar to Glyoxalase I.
Os05g0231600	Similar to Aquaglyceroporin (Tonoplast intrinsic protein (Tipa)).
Os05g0231700	Similar to Tonoplast membrane integral protein ZmTIP4-2.
Os05g0231900	Similar to Peroxidase 29 precursor (EC 1.11.1.7) (Atperox P29) (ATP40).
Os05g0232500	Ubiquitin-conjugating enzyme, E2 domain containing protein.
Os05g0233900	Zinc finger, TTF-type domain containing protein.
Os05g0234700	Zinc finger, TTF-type domain containing protein.
Os05g0235300	Armadillo-like helical domain containing protein.
Os05g0235800	MCM protein 6 family protein.
Os05g0237100	Similar to Type A-like cyclin.
Os05g0237300	Non-protein coding transcript, unclassifiable transcript.
Os05g0237400	Similar to Viroid symptom modulation protein.
Os05g0237700	Terpenoid cylases/protein prenyltransferase alpha-alpha toroid domain containing protein.
Os05g0238200	Similar to Ubiquitin ligase SINAT5 (EC 6.3.2.-) (Seven in absentia homolog 5).
Os05g0238400	Harpin-induced 1 domain containing protein.
Os05g0239200	Non-protein coding transcript, unclassifiable transcript.

Os05g0240200	Similar to CTV.2.
Os05g0241000	Prefoldin domain containing protein.
Os05g0241100	Similar to Leucyl-tRNA synthetase, cytoplasmic (Leucine--tRNA ligase) (LeuRS).
Os05g0241200	Similar to Phophate translocator (Fragment).
Os05g0241900	Retrotransposon gag protein family protein.
Os05g0242000	Protein of unknown function DUF247, plant family protein.
Os05g0242600	Prefoldin domain containing protein.
Os05g0243200	Similar to Ribosomal protein L13.
Os05g0243300	Similar to 50S ribosomal protein L13.
Os05g0244500	Glycoside hydrolase, family 5 protein.
Os05g0244600	Nucleic acid-binding, OB-fold domain containing protein.
Os05g0244700	Aminotransferase, class IV family protein.
Os05g0245300	Protein of unknown function DUF588 family protein.
Os05g0246300	Expansin/Lol pI family protein.
Os05g0246600	Protein kinase domain containing protein.
Os05g0247100	Similar to Chitinase (EC 3.2.1.14) III C00481-rice (EC 3.2.1.14).
Os05g0247500	Glycoside hydrolase, family 18 protein.
Os05g0247600	RNA-directed DNA polymerase (Reverse transcriptase) domain containing protein.
Os05g0247800	Glycoside hydrolase, family 18 protein.
Os05g0247900	DEAD/DEAH box helicase, N-terminal domain containing protein.
Os05g0248200	Glycoside hydrolase, family 18 protein.
Os05g0250700	Ribosomal protein L13 family protein.
Os05g0251500	Similarities with spP29295 Saccharomyces cerevisiaeYPL204w HRR25 casein kinase I.
Os05g0251900	Oligopeptide transporter OPT superfamily protein.
Os05g0252000	Oligopeptide transporter OPT superfamily protein.
Os05g0252100	Protein of unknown function DUF620 family protein.
Os05g0253200	Protein kinase-like domain containing protein.
Os05g0255600	Thioredoxin domain 2 containing protein.
Os05g0256000	Similar to TGF-beta receptor-interacting protein 1.
Os05g0256100	Serine/threonine protein kinase domain containing protein.
Os05g0256500	Protein kinase domain containing protein.
Os05g0257100	Leucine-rich repeat, plant specific containing protein.

Table S4. List of primers used in this study.

Name	Forward primer (5′-3′)	Reverse primer (5′-3′)
OsIAA17q5	TGAAGAGCAGCAAGGAGGAT	CGGCTCTCAGATAAGCCATC
OsActin	CCCTCCTGAAAGGAAGTACAGTGT	GTCCGAAGAATTAGAAGCATTTCC
